# Magnetic resonance image-guided adaptive radiotherapy enables safe CTV-to-PTV margin reduction in prostate cancer: a cine MRI motion study

**DOI:** 10.3389/fonc.2024.1379596

**Published:** 2024-06-04

**Authors:** Rosalyne L. Westley, Sophie E. Alexander, Edmund Goodwin, Alex Dunlop, Simeon Nill, Uwe Oelfke, Helen A. McNair, Alison C. Tree

**Affiliations:** ^1^ Department of Radiotherapy, The Royal Marsden NHS Foundation Trust, London, United Kingdom; ^2^ Division of Radiotherapy and Imaging, The Institute of Cancer Research, Sutton, United Kingdom; ^3^ Joint Department of Physics, The Institute of Cancer Research and The Royal Marsden NHS Foundation Trust, Sutton, United Kingdom

**Keywords:** MRIgART, CTV-PTV margins, prostate, ultra-hypofractionation, SBRT, intrafraction motion

## Abstract

**Introduction:**

We aimed to establish if stereotactic body radiotherapy to the prostate can be delivered safely using reduced clinical target volume (CTV) to planning target volume (PTV) margins on the 1.5T MR-Linac (MRL) (Elekta, Stockholm, Sweden), in the absence of gating.

**Methods:**

Cine images taken in 3 orthogonal planes during the delivery of prostate SBRT with 36.25 Gray (Gy) in 5 fractions on the MRL were analysed. Using the data from 20 patients, the percentage of radiotherapy (RT) delivery time where the prostate position moved beyond 1, 2, 3, 4 and 5 mm in the left-right (LR), superior-inferior (SI), anterior-posterior (AP) and any direction was calculated.

**Results:**

The prostate moved less than 3 mm in any direction for 90% of the monitoring period in 95% of patients. On a per-fraction basis, 93% of fractions displayed motion in all directions within 3 mm for 90% of the fraction delivery time. Recurring motion patterns were observed showing that the prostate moved with shallow drift (most common), transient excursions and persistent excursions during treatment.

**Conclusion:**

A 3 mm CTV-PTV margin is safe to use for the treatment of 5 fraction prostate SBRT on the MRL, without gating. In the context of gating this work suggests that treatment time will not be extensively lengthened when an appropriate gating window is applied.

## Introduction

The 1.5 T MR Linac (MRL) (Elekta, Stockholm, Sweden) integrates a Philips 1.5 T magnetic resonance imaging (MRI) scanner (Best, The Netherlands) with an Elekta 7-MV linear accelerator (Stockholm, Sweden) to offer real-time MRI with online adaptive radiotherapy (RT) delivery (1, 2).

MRI is the gold standard modality for imaging of the pelvis, as such MRI-guided prostate cancer (PCa) RT allows for precise contouring and accurate online matching (3). Furthermore, the adaptive capabilities of the MRL (4, 5) allows the treating team to account for observed interfraction motion and deformation of the prostate, seminal vesicles, and associated organs at risk (OARs) that may occur over a course of treatment (6–11). Prostate motion occurring during the planning stage, whilst the patient lies on the couch, can also be accounted for. Combined, these features mitigate contouring and motion errors factored into clinical target volume (CTV)-to-planning target volume (PTV) margin calculations potentially enabling PTV margin reduction (12).

Smaller margins result in less dose to surrounding normal tissue, with the potential to reduce RT toxicity in tumour groups such as the prostate. The MIRAGE trial tested this hypothesis in a single-centre phase III clinical trial where men received SBRT with either CT-guided RT (CTgRT) with a 4-mm CTV-to-PTV margin or MRI-guided adaptive RT (MRIgART) with a 2-mm CTV-to-PTV margin (13). MIRAGE reported a significant reduction in acute grade ≥2 genitourinary (GU) toxicity with MRI versus CT guidance (24.4% vs. 43.4%, *p* = 0.01) and a similarly significant reduction in acute grade ≥2 gastrointestinal (GI) toxicity (0% vs. 10.5%, *p* = 0.003). Daily recontouring was not performed in MIRAGE; however, gating was initiated if 10% of the prostate volume moved outside of a 3-mm gating boundary (14).

Gating describes the process whereby an automatic beam hold is initiated if the target moves outside of a pre-defined threshold. Baseline shift correction describes the movement of the beam to the new target position if it moves from its initial position. These correction strategies are not currently available on our 1.5T MRL but are included with the upcoming upgrade to comprehensive motion management (CMM) (15).

With the move to treating more men with SBRT on the MRL, whilst simultaneously aiming to limit toxicity, it seems prudent to test the concept of margin reduction as adopted in MIRAGE (13, 16). If smaller margins than those currently adopted were to show adequate prostate coverage, this could lead to a reduction in margins from the current standard (5 mm except 3 mm posteriorly) (16), with the aim of improving toxicity whilst maintaining good cancer control.

Novel images acquired during RT beam-on time were utilised to assess the suitability of a range of CTV-to-PTV margins. These margins were tested in the context of MRIgART, with daily treatment adaption and no gating software.

## Materials and methods

The RT image datasets of 20 patients with PCa treated radically on a 1.5T MRL at The Royal Marsden NHS Foundation Trust were retrospectively reviewed and analysed. All patients received 36.25 Gy in five fractions with a CTV-to-PTV margin of 5 mm and 3 mm posteriorly applied (16). Patients were recruited to PERMIT (NCT03727698) and MOMENTUM (NCT04075305), consenting to the use of their imaging for research (17, 18).

### Offline workflow

Prior to treatment, a planning computed tomography (CT) (Siemens Confidence, Munich, Germany) scan and a planning MRI on a 1.5 T diagnostic scanner (Siemens Aera, Munich, Germany) were acquired within 2 h of each other. Patients were prescribed microlet enemas 2 days before and on the day of scanning and instructed to drink 350 mL of water 60 min before their CT to achieve a comfortably full bladder volume. Bladder preparation was repeated prior to the planning MRI. Patients were positioned head-first supine, with indexed knee and foot immobilisation and a head support.

The CT and MR images were registered for contouring using soft tissue, focusing on the prostate and surrounding tissues, in the RayStation treatment planning system (TPS) (Raysearch Laboratories, Stockholm, Sweden, V8.0.0.61) (5). The offline MRL reference plan was generated using the Monaco TPS (Elekta AB, Stockholm, Sweden, V5.40.00) (5, 19).

### Online workflow

Bladder preparation, bowel preparation, and immobilisation were as per the offline workflow. Patients were positioned by two therapeutic radiographers (RTTs) aligning tattoos with the sagittal laser and referenced couch index position (20, 21). A T2-weighted 3-Dimensional Transverse MRI (T23DTra), with a 1-mm slice thickness, was acquired for daily replanning, referred to as MRI_session_. This image was registered, based on prostate soft tissue, to the reference image and an adapt-to-shape (ATS) workflow followed (19). A second T23DTra MRI was acquired at the latter stages of plan optimisation to confirm prostate position before treatment, referred to as MRI_verification_. If the prostate had moved outside of the PTV margin, an ATP workflow of the daily ATS plan was applied to correct for prostate translational deviations seen on the MRI_verification_ (5). On completion of dose delivery, a post-treatment T23DTra MRI, referred to as MRI_post,_ was taken.

Continuous motion monitoring (MM) was started 30–60 s prior to beam on, using a 3D balanced turbo field echo (bTFE) sequence to produce single-slice cine images in three orthogonal planes. Prostate position relative to the PTV structure was monitored by the RTTs during treatment delivery. If the prostate breached the PTV contour, treatment was interrupted until the prostate moved back within the margin, thus acting as a manual gating tool. MM was stopped on completion of treatment.

### Cine motion measurement

Cine images were obtained with an average frequency of every 0.620 s for MM during treatment. The motion occurring between each cine frame was calculated using an optical flow method (22–24). A cine frame at the start of the acquisition was used as a reference, and an ROI was defined on the reference cine by propagating contours from the daily structure set. Subsequent cine images were registered to the reference, and the average motion vector of the ROI was calculated. Offsets from orthogonal planes for each axis were averaged to derive a single shift in each plane, e.g., lateral offsets measured on the transverse and coronal cine images were averaged to give a single lateral shift per frame. This automated method was previously validated against prostate centroid motion calculated from prostate volumes manually contoured by two clinically experienced and competency-approved practitioners (RW and SA).

The algorithm was run on the cine imaging acquired during MM for every fraction of all 20 patients, giving the displacement of the prostate CTV in three orthogonal planes. For each fraction, the number of time points where the prostate position moved beyond 1, 2, 3, 4, and 5 mm in the left–right (LR), superior–inferior (SI), anterior–posterior (AP), and any direction was calculated. From these data, the total percentage of the MM time that the prostate spent within the above margins was determined.

The percentage of patients whose prostate spent 90% or more of the monitoring time within 1, 2, 3, 4, and 5 mm of the starting position was established. The difference between the three orthogonal planes was compared using the Kruskal–Wallis test (GraphPad Prism 10). Coverage was deemed to be acceptable if the margin allowed for 95% of the patients to spend 90% of their treatment time within it. Because of the outcomes of MIRAGE and the absence of gating, this paper focuses on 2-mm and 3-mm margins. The percentage of the number of fractions within a 2-mm and 3-mm margin for 90% of the MM time was also calculated.

The patterns of motion observed by the prostate in each direction were reviewed by plotting motion against time for each fraction of RT. These were grouped together for each patient to allow better visualisation of an individual’s prostate motion. The corresponding anatomy as seen on the cine imaging was reviewed to help determine patient factors that could account for the motion seen.

## Results

### Patient cohort

Twenty consecutive patients who received radical prostate RT prior to May 2021 were included. Mean age was 72 years (SD = 6.48), with a median PSA of 6.55 (range: 2–12.6). Nineteen (95%) patients had T2 disease and one (5%) had T3a disease. Gleason score was 3 + 3 (5%), 3 + 4 (80%), and 4 + 3 (15%).

For each patient, all five RT fractions were delivered on the MRL following an ATS workflow. Twenty-eight (28%) fractions required an additional ATP correction based on the position of the prostate on the MRI_verification_. One patient had one of their fractions terminated early due to a beam delivery fault. The remainder of the fraction was delivered with an additional ATP-only workflow. During treatment, one patient had their RT paused due to coughing whilst on the couch, and MM continued to run during this period.

### Treatment timings

The median time from the MRI_session_ to the MRI_verification_ was 30 min (range: 8–45 min) with the median time from the MRI_verification_ to the MRI_post_ being 16 min (range: 8–59 min). Prostate motion was evaluated on the MM acquired during 100 PCa RT fractions. The median time of MM during treatment was 9.73 min per fraction and 44.63 min for a course of five fractions.

### Prostate motion

In 65% of patients, the prostate moved less than 2 mm in any direction for 90% of the MM period. For a further 30% of patients, the prostate moved less than 3 mm for 90% of the MM period. This equates to the prostate moving less than 3 mm in any direction for 90% of the monitoring period in 95% of patients. **Table 1** shows the percentage of patients who spent 90% of the monitoring period within a 1-, 2-, 3-, 4-, and 5-mm margin.

**Table 1:** Percentage of patients with their prostate staying within a 1-, 2-, 3-, 4-, and 5-mm margin for 90% of the total MM time in each plane and in all directions.

	Percentage of patients			
	LR	SI	AP	All directions
**90% of monitoring time within 1 mm**	85%	40%	20%	10%
**90% of monitoring time within 2 mm**	100%	80%	75%	65%
**90% of monitoring time within 3 mm**	100%	95%	95%	95%
**90% of monitoring time within 4 mm**	100%	95%	100%	95%
**90% of monitoring time within 5 mm**	100%	100%	100%	100%

In the LR direction, 100% of patients moved less than 2 mm for 90% of the monitoring period, compared to 80% in the SI direction and 75% in the AP direction. The time spent outside of 2 mm was significantly less in the LR direction than the other directions (*p* = 0.0014).

On a per-fraction basis, 77 fractions (77%) had motion within a 2-mm margin in all directions for 90% of the fraction MM time. Ninety-three fractions (93%) displayed motion in all directions within 3 mm for 90% of the fraction MM time.

**Figure 1:**
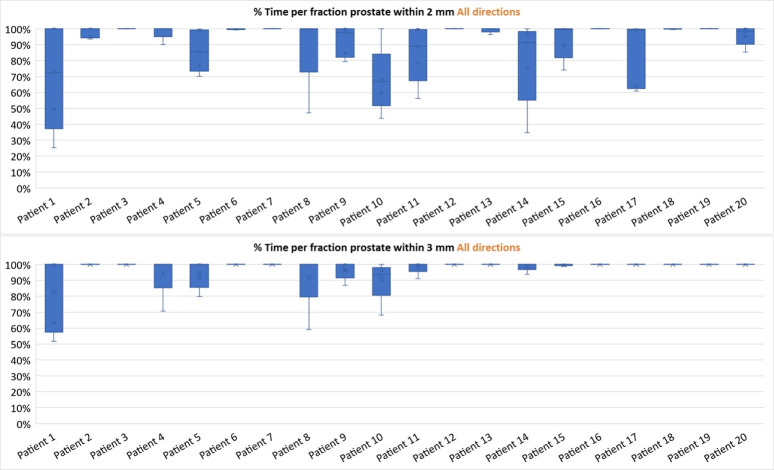
Box-and-whisker plots showing the spread of the percentage of motion monitoring (MM) time within 2 mm (top) and 3 mm (bottom) for each fraction separated into individual patients.

**Figure 1** shows the percentage of monitoring time that each fraction spent within 2 mm and 3 mm for each patient. Some patients (e.g., patient 3) have minimal motion seen through all five fractions, whilst other patients (e.g., patient 1) display a wider range of motion across all fractions.

### Type of motion observed and patient factors

**Figure 2:**
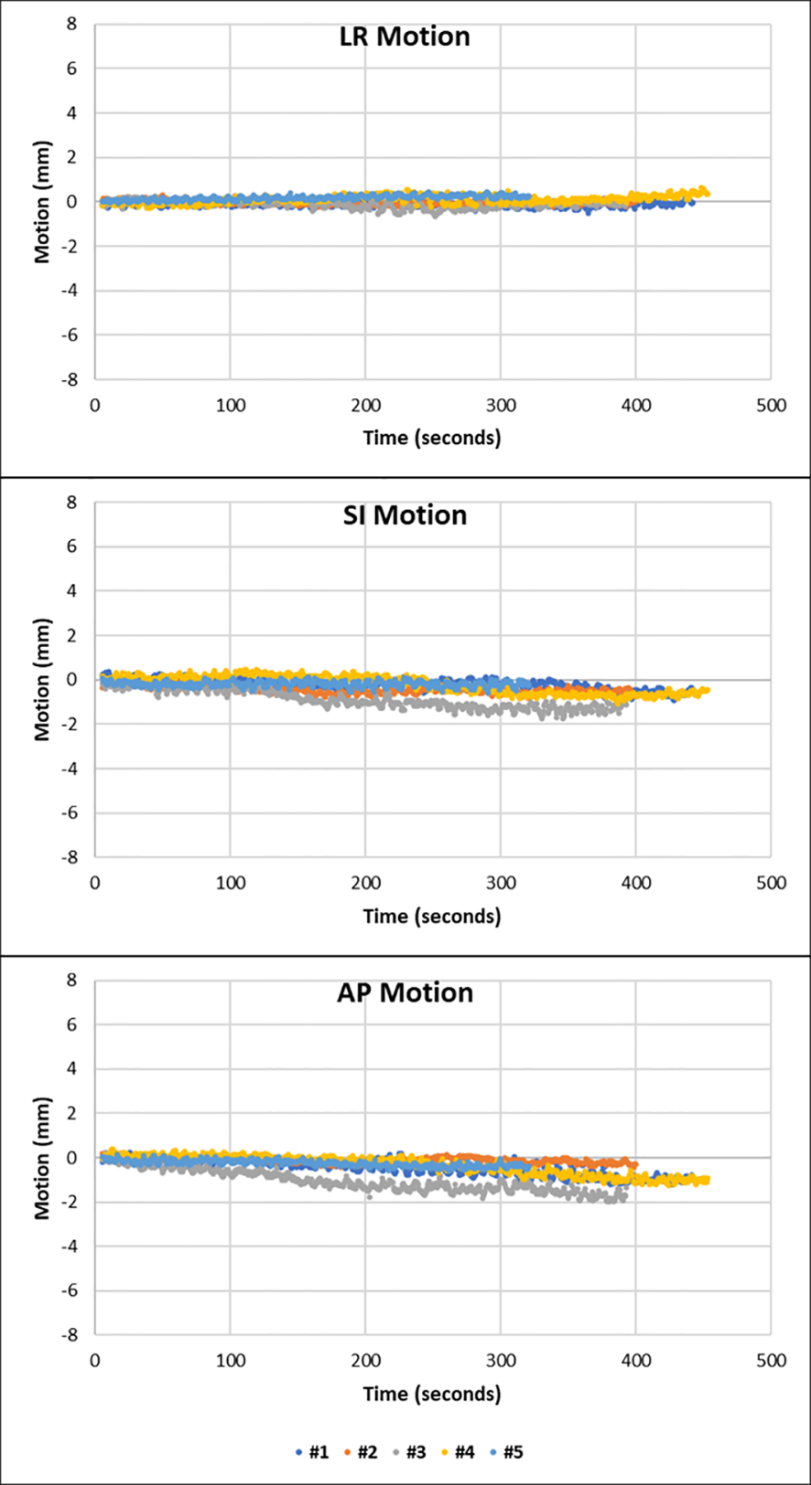
Motion graphs to show the motion observed against time in the LR, SI, and AP direction of each fraction from patient 7. There is minimal movement in the LR direction with shallow drift shown in the inferior and posterior direction.

There were three distinct patterns of prostate motion observed throughout this study. Most fractions displayed a shallow continuous drift, with the prostate drifting in a continuous direction with an amplitude of no more than 2 mm in any direction from the position at the start of monitoring. The motion graphs for each patient are presented in **Supplementary Figures S1A–C**. **Figure 2** shows the motion in the LR, SI, and AP direction of all five fractions from patient 7. This patient is shown as in each fraction the prostate moves with shallow continuous drift with the prostate moving inferiorly and posteriorly during MM. This motion was less than 2 mm from start of treatment in all three planes.

The MRI_verification_ and MRI_post_ of each fraction from patient 7 show that the position and size of the rectum and bladder remain relatively stable between the start and end of treatment (**Supplementary Figure S2**). Furthermore, there is no acute change in the size or position of the anatomy seen on the MM.

**Figure 3:**
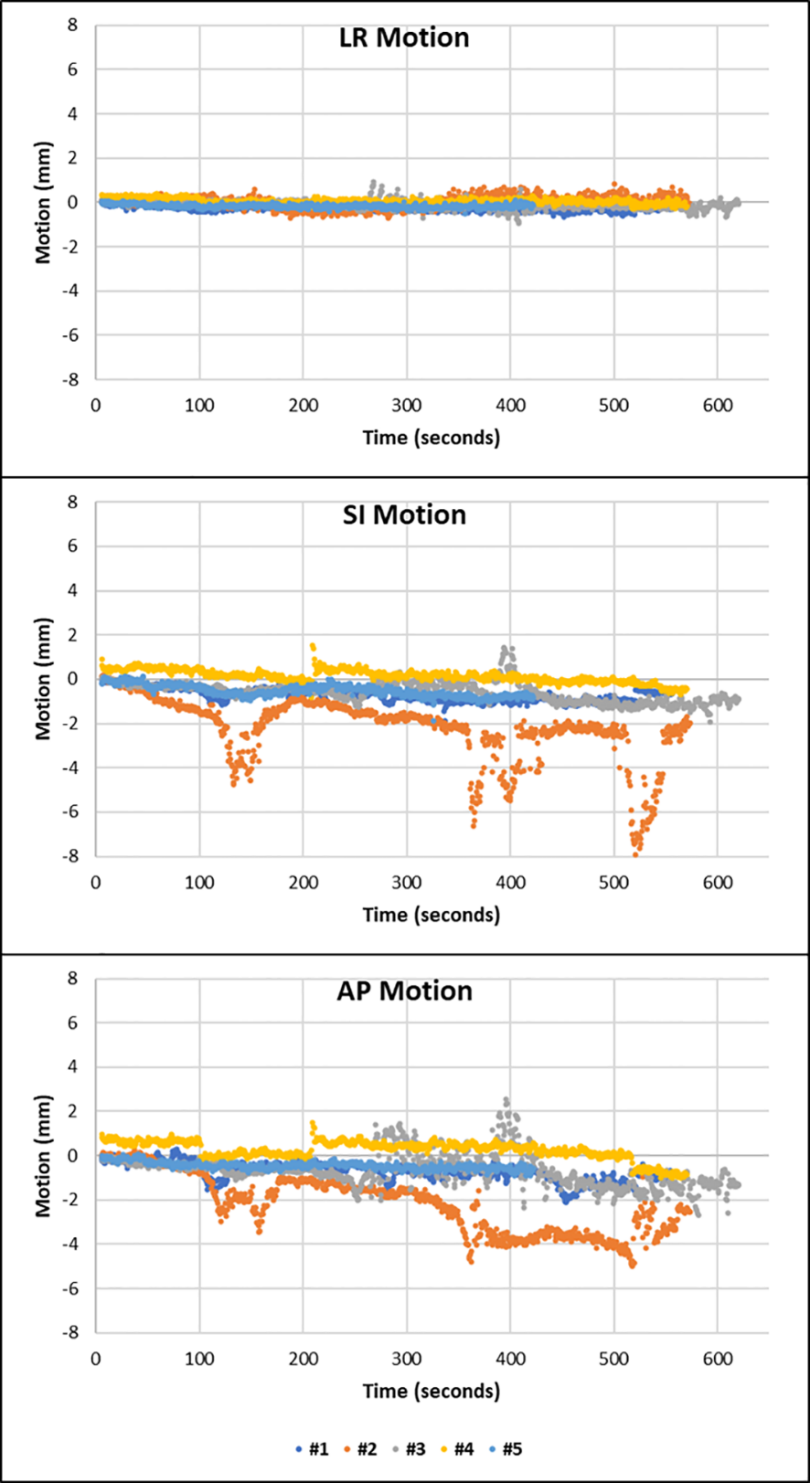
Patient 8 displayed drift motion with transient excursions in fractions 2 and 3. Transient excursions are seen in the SI and AP direction.

MM showed that five patients had at least one or more fractions in which the prostate displayed transient excursions on top of the drift pattern. This can be seen in patient 8, where the motion is such that the prostate follows this pattern in fractions 2 and 3 (**Figure 3**).

The passage of air and faeces through the rectum during treatment delivery can be observed on cine imaging along with the impact this has on the rectal diameter and the resultant shift in the position of the prostate. Such motion explains the cause of the large amplitudes of motion seen in fraction 2 of patient 8 in **Figure 3** and is demonstrated in **Supplementary Figure S3**.

**Figure 4:**
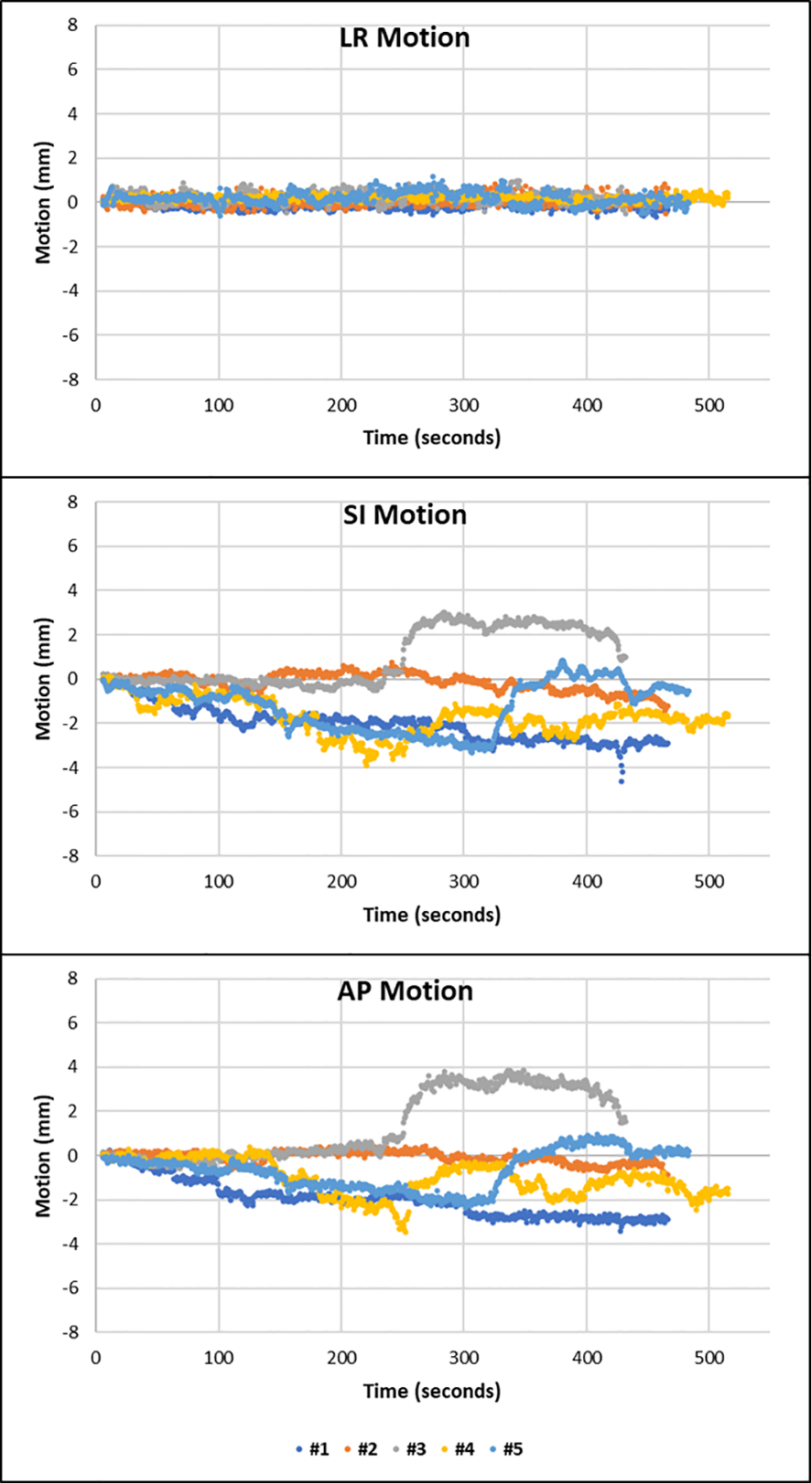
Fractions 2 and 5 of patient 10 showing that the prostate suddenly deviates away from the general trend of motion to a different position. The prostate does not fall back to its initial position. This is shown in the SI and AP direction.

The third pattern observed was that of sudden erratic single points of motion with persistent excursion of the prostate to a new position, away from the general trend of motion. This was observed in at least one fraction of three patients. Patient 10 displays this motion in the AP and SI directions in fractions 2 and 3 (**Figure 4**).

MM imaging showed how the patient clenches their buttocks during fraction 3, with their whole pelvic anatomy shifting superiorly. This is represented in the cine image in **Supplementary Figure S4**.

## Discussion

This work shows that for 95% of patients, the prostate will be within a 3-mm margin for 90% of the treatment monitoring time. Such results suggest that 3 mm would be a safe CTV-to-PTV margin when treating men with PCa on a 1.5T MRL in the absence of CMM. Gating is already a standard feature of the 0.35-T MRL (ViewRay MRIdian, ViewRay Inc., Cleveland, OH, USA) and is currently being implemented on the 1.5T platform, the implications of which are discussed below.

Non-isotropic margins with 2 mm in the LR direction and 3 mm in the AP and SI direction could be justified. This concept has also been demonstrated on earlier cine and electromagnetic tracking data (25, 26). A reduction in lateral margins may help to decrease the dose to lateral structures including the neurovascular bundles and internal pudendal artery, which is hypothesised to improve sexual dysfunction, the most prevalent long-term side effect of PCa RT (27, 28).

The cutoff of 90% of the treatment time was chosen using the Van Herk et al. concept for deriving margins from dose-population histograms to ensure a minimum dose to the CTV of 95% for 90% of patients (29). With MRL treatment, we are not looking at ensuring CTV coverage for 90% of patients, as the PTV concept was originally intended, but instead looking at ensuring coverage 90% of the time for each patient. As no margin covers 100% of the patients, a margin covering 95% of the patients, 90% of the time, was considered acceptable.

Prostate motion trends seen on MM align with previous work. The Calypso 4D Localization System (Calypso System, Calypso Medical. Seattle, WA) showed six patterns of motion ranging from “stable target at baseline” to “erratic behaviour” (30). The patterns demonstrated by this patient cohort were also observed by Kupelian et al. (2007) (30); “continuous target drift” described the pattern shown by patient 7, “transient excursions” described the motion shown by patient 8, whilst “persistent excursions” describes the motion shown by patient 10 (30). It is equally important to characterise the pattern of intrafraction prostate motion in conjunction with the amplitude of motion observed as this can have dosimetric and therefore clinical implications.

Shallow drift of the prostate was seen in the majority of fractions. For these fractions, a 2-mm margin could be considered acceptable, but unfortunately, there is currently no pre-treatment biomarker to predict which patients will show larger amplitudes of drift or more significant excursions during any one fraction (31), a concept supported in the work by Litzenber et al. (2006) (26). Optimal practice would be to predict patients who are likely to show larger patterns of motion and apply individual margins to these patients.

At present, our 1.5T MRL allows the treating RTT to pause RT treatment whilst the patient is on the couch, thus acting as a manual gating system when larger transient excursions might be observed. However, this system is not sensitive enough to warrant safely reducing CTV–PTV margins down to 2 mm. This method also relies on the prostate falling back to its original position for the remainder of the fraction to be to be delivered.

The conclusions drawn in this study are based on the prostate intrafraction motion measured during MM. Yet, in practice, prostate motion may occur during the period between the MRI_session_ and MRI_verification_ and between MRI_verification_ and start of MM. To minimise the impact any further motion could have on dosimetry, an ATP shift was performed if the CTV moved outside of the PTV as seen on the MRI_verification_. In HERMES (NCT04595019), a novel trial randomising between five-fraction and two-fraction MRIgART to the prostate, a 3-mm CTV–PTV margin was adopted and an ATP workflow was instigated for all but negligible prostate motion seen on the MRI_verfication_ (32). We would recommend this workflow if moving to a 3-mm CTV–PTV margin in the absence of gating.

The additional ATP workflow accounts for any motion prior to the MRI_verification_ but does not correct for motion occurring after (4). Reassuringly, the impact of intrafraction motion occurring after the MRI_session_ with an ATS workflow has shown that a 3-mm margin provided sufficient prostate coverage (33). MM started after the verification scan would help to clarify the clinical relevance of this motion and any impact it may have; indeed, as the prostate moves less than 2 mm in many cases or only transiently moves outside 2 mm, it is unlikely to have a significant impact.

The motion algorithm used in this study measures motion akin to the method that will be used on a 1.5 T MRL once CMM is introduced (34). Once gating and baseline shift correction have been implemented, further margin reduction as adopted by MIRAGE could be considered (13). The characterisation of prostate motion has highlighted the need for both baseline correction and gating if a further reduction in margins is to be employed. Whilst gating could be deployed in transient excursions, it would be futile in patients displaying continuous target drift and persistent excursions (30). Baseline shift corrections would allow the treating team to account for prostate motion occurring between the planning phase and MM and for persistent drift or excursion when the beam is on. This study provides reassurance that margin reduction in the presence of gating, such as already utilised clinically on a 0.35-T MRL, will not interrupt treatment in most fractions, when a 2-mm gating window is applied, keeping treatment disruption and delivery time to a minimum for MRIgART.

Despite the conclusion that a 3-mm margin adequately accounts for motion, the fundamentals of clinical impact should be considered, especially when aiming to reduce toxicity such as shown in MIRAGE (13). A 2-mm margin could be considered a clinically realistic PTV margin with the concept that small breaches of a 2-mm PTV margin (<1 mm) may have little impact on the dose required for a cure (4, 29). This concept is currently being tested in DESTINATION (NCT05709496), a feasibility study that aims to decrease the dose to the OARs whilst maintaining good cancer outcomes in the context of MRIgART. In this trial, men receive dose-reduced SBRT to the prostate with no CTV–PTV margin and a simultaneous integrated boost to the GTV+4 mm (35). This trial considers the possibility that not all of the prostate requires treatment to a dose needed for ablating macroscopic disease (35). Even despite this concept, preliminary work suggests that sufficient coverage to the whole prostate will be met even when a PTV margin is not applied due to the adaptive capabilities of the MRL and the nature of the dose fall off when boosting the MRI visible tumour to 45 Gy (36). The use of CMM will support the safety of such a strategy if the results show favourable toxicity outcomes.

When applying the findings of this study to the clinical workflow, the treating team must be mindful of the planning MRI parameters used and how their TPS manages margin expansion. Prior work by Chick et al. (2023), which evaluated non-vendor sequences on the MRL using the Monaco TPS as used in this study, showed how using PTV margins that are non-integer multiples of the slice thickness can cause the effective TPS margins to differ superiorly and inferiorly from what was intended (37). In centres where the slice thickness of the planning MRI is greater than 1 mm, the impact that this may have on margin behaviour must be evaluated with an end-to-end assessment of the workflow. As the planning MRIs used in our centre for this protocol have a slice thickness of 1 mm, we are confident that the 3 mm stipulated can be applied accurately.

This study has limitations recognised by the authors, and the potential intrafraction motion prior to the first cine image has been addressed previously. The size of this study could be considered a study limitation as a total 20 patients were included in the analysis. Although the results were primarily interpreted on a per-patient basis, as this was felt to be more clinically applicable, each patient had five fractions with all of them contributing to the results of the study. The motion data from each of the 100 fractions were also analysed separately, with the results in agreement to those found on a per-patient basis. By analysing 100 fractions, the random error has been suitably reduced (38).

In the era of MRIgART, the resultant improvement in the accuracy of daily matching and contouring has resulted in a move away from applying a value for contouring error in the CTV-to-PTV margin calculation (13, 32). Despite contouring studies having shown that MRI-based contouring is significantly superior to CT-based contouring, it remains a limitation of this study that the impact of contouring variation was not addressed (39, 40). We remain optimistic that there would be little resultant impact on prostate coverage and indeed the dose to the CTV (41).

## Conclusion

A 3-mm CTV-to-PTV margin seems to be a safe treatment option for MRIgART, in the absence of gating and baseline correction. The application of gating and baseline correction may promise to facilitate a further reduction in CTV–PTV margins without a meaningful extension in treatment time. In the meantime, it is prudent to continue to search for parameters that identify patients displaying larger amplitudes or persistent intrafraction motion, which would enable personalised CTV-to-PTV margins.

## Data availability statement

The original contributions presented in the study are included in the article/**Supplementary Material**. Further inquiries can be directed to the corresponding author.

## Author contributions

RW: Conceptualization, Data curation, Formal analysis, Investigation, Methodology, Project administration, Resources, Validation, Visualization, Writing – original draft, Writing – review & editing. SA: Conceptualization, Data curation, Formal analysis, Investigation, Methodology, Project administration, Resources, Validation, Visualization, Writing – original draft, Writing – review & editing. EG: Data curation, Investigation, Methodology, Software, Writing – review & editing. AD: Writing – review & editing. SN: Writing – review & editing. HM: Supervision, Writing – review & editing. AT: Supervision, Writing – review & editing, Conceptualization. UO: Writing – review & editing, Supervision.
