# Supplementary material for: Magnetic resonance image-guided adaptive radiotherapy enables safe CTV-to-PTV margin reduction in prostate cancer: a cine MRI motion study
Source: Front Oncol. 2024 Jun 4;14:1379596. doi: 10.3389/fonc.2024.1379596 (PMC11183304; doi:10.3389/fonc.2024.1379596)
Supplement: Supplementary file 1 [file DataSheet_1.docx]

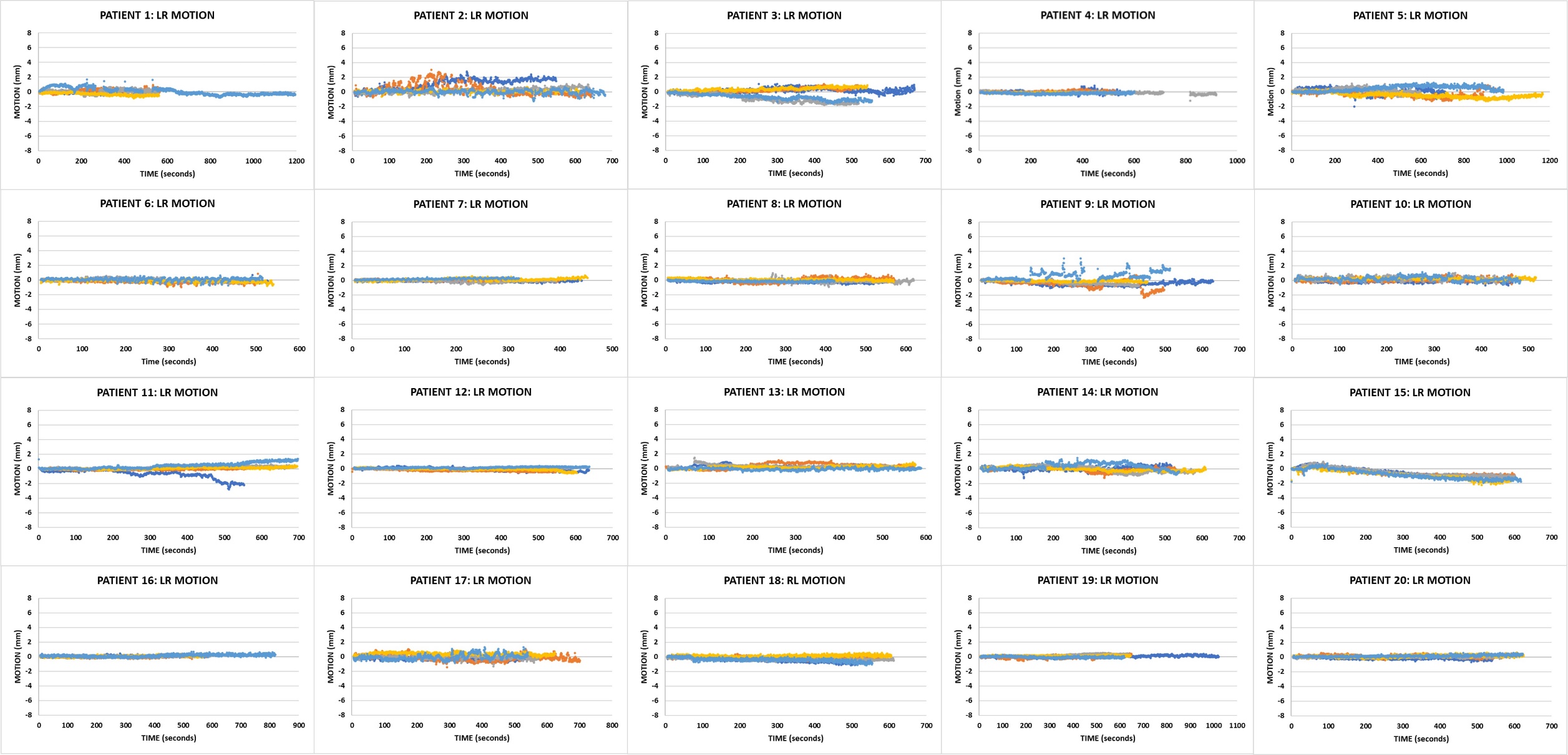


*S1a.* Motion in the left/right direction for all patients for all five fractions. #1 = dark blue, #2 = orange, #3 = grey, #4 = yellow, #5 = light blue.


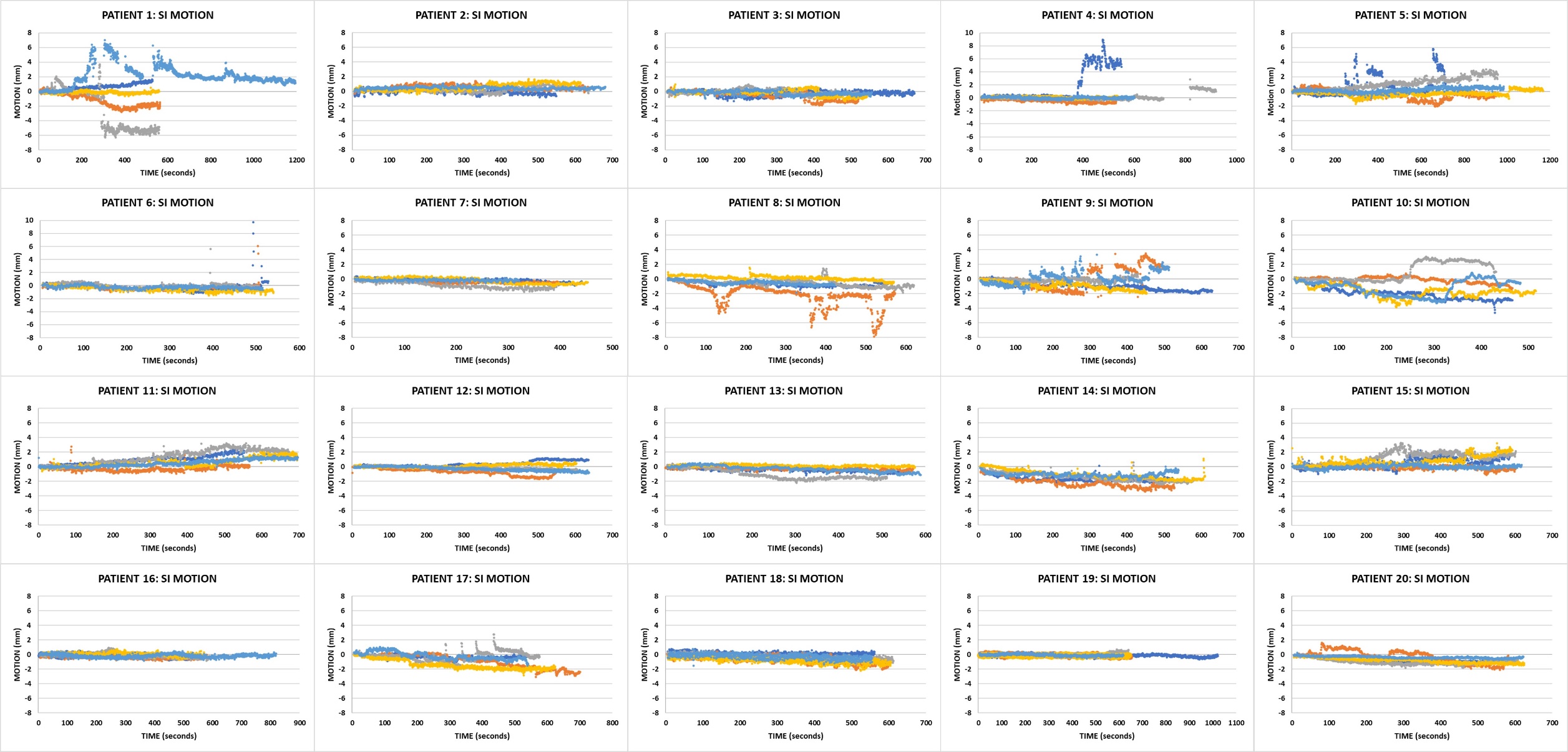


*S1b.* Motion in the superior/inferior direction for all patients for all five fractions. #1 = dark blue, #2 = orange, #3 = grey, #4 = yellow, #5 = light blue.


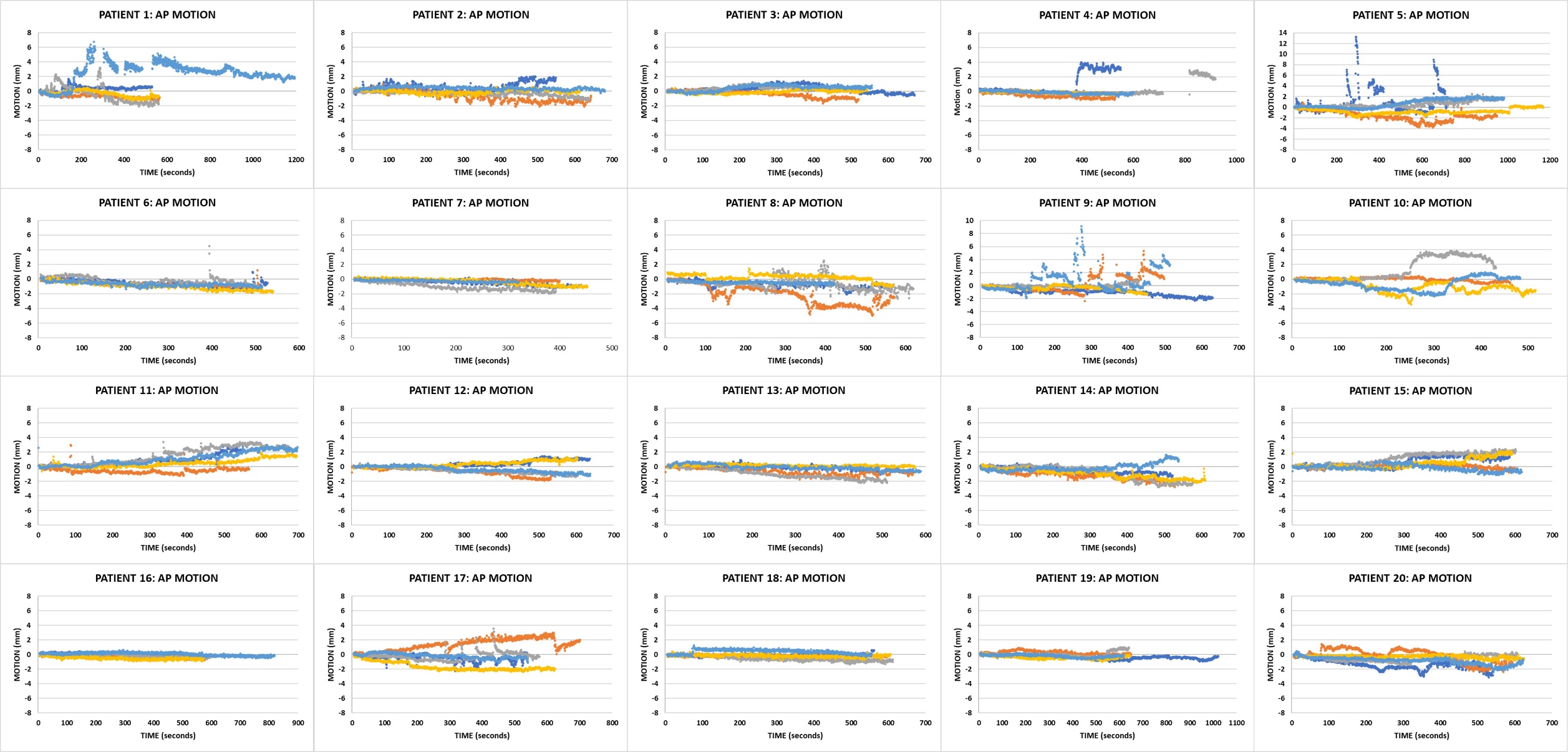


*S1c.* Motion in the anterior/posterior direction for all patients for all five fractions. #1 = dark blue, #2 = orange, #3 = grey, #4 = yellow, #5 = light blue.


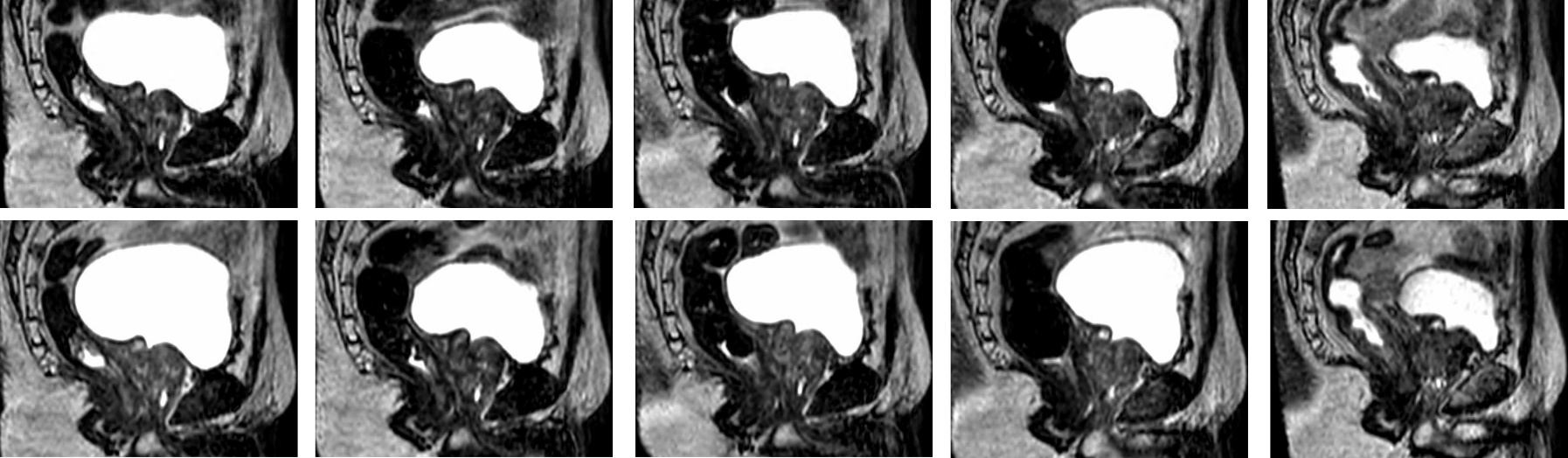


S2. The MRI_verification_ (upper level) and MRI_post_ (lover level) show the prostate, bladder and rectum before and after treatment for **patient 7**. There is no obvious changes in anatomy which would be expected due to the gentle drift pattern seen in the position of the prostate.

**
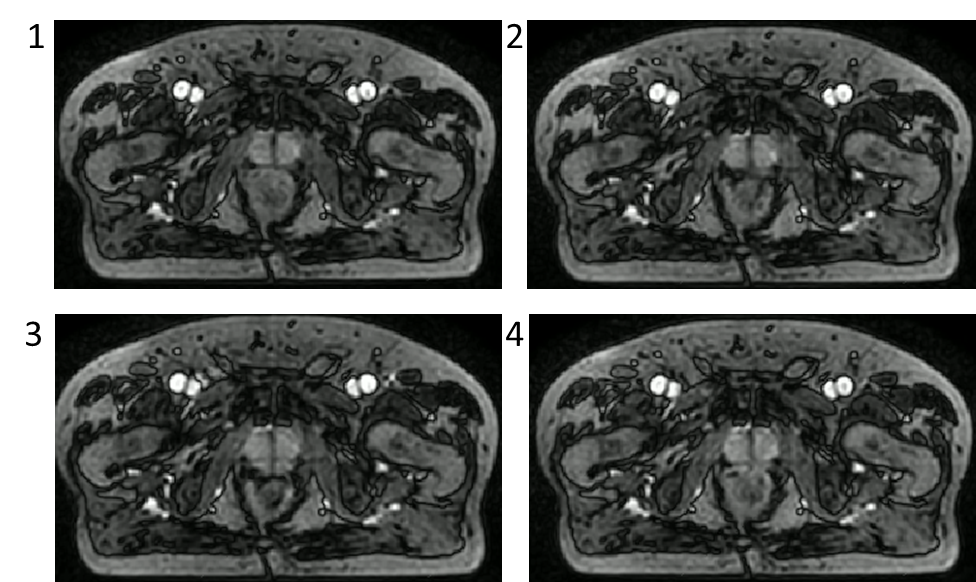
**

S3. The cine images of patient eight shown in chronological order, demonstrating the narrowing and then filling of the rectum during fraction 2. This occurred three times during the motion monitoring (MM) explaining the transient excursions seen on the graph (**Figure 3**).


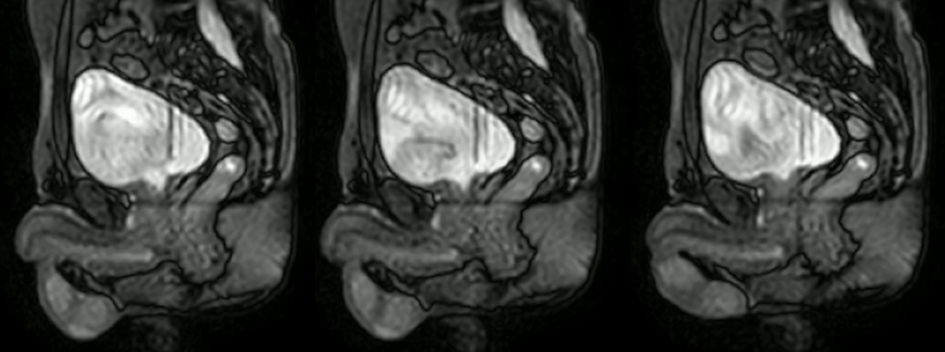


S4. Cine images taken during motion monitoring show patient 10 clenching and their prostate moving superiorly during fraction 3. This accounts of the movement of the prostate shown in **Figure 4**.
